# Does Perceiving the Poor as Warm and the Rich as Cold Enhance Perceived Social Justice? The Effects of Activating Compensatory Stereotypes on Justice Perception

**DOI:** 10.3389/fpsyg.2019.01361

**Published:** 2019-06-18

**Authors:** Anguo Fu, Zaisheng Zhang, Wuming He, Zhaohong Lin, Na Wu, Guanghui Hou, Tianzeng Yao

**Affiliations:** ^1^College of Management and Economics, Tianjin University, Tianjin, China; ^2^School of Management, Hainan University, Haikou, China; ^3^School of Educational Science, Lingnan Normal University, Zhanjiang, China; ^4^Department of Tourism Management, Hainan College of Economics and Business, Haikou, China

**Keywords:** compensatory stereotypes, stereotype content model, personal justice perception, systemic justice perception, system justification motive

## Abstract

Compensatory stereotypes are the fundamental components of social perception, and competence and warmth are the two fundamental dimensions of social cognition. Previous studies have concluded that, to maintain belief in justice, the system justification motive leads people to believe that upper- and lower-class groups each have their own unique and mutually offsetting advantages and disadvantages (e.g., the rich have low warmth and the poor have high warmth). The present study introduced the variable of social justice perception (personal and systemic justice perception) and hypothesized that endowing upper-class groups with negative characteristics and lower-class groups with positive characteristics could enhance people’s social justice perception. Participants were presented with vignettes that activated compensatory/non-compensatory stereotypes in four ways (compensatory competence, non-compensatory competence, compensatory warmth, non-compensatory warmth) regarding individuals described as rich and poor. Justice perception toward these individuals was then rated by the participants. The results showed that compensatory stereotypes triggered by system justification motives can affect the social justice perceptions of individuals to a certain extent. That is, perceiving the poor as warm and the rich as cold enhances perceived social justice, whereas perceiving the poor as competent and the rich as incompetent reduces perceived personal justice but does not affect perceived systemic justice. Especially in the context of the Chinese Confucian culture, which emphasizes warmth but ignores competence, the effect of compensatory stereotypes on perceptions of social justice underscores a cultural difference with the West that warmth is superior to competence. Further, compensatory stereotypes may be either beneficial for or detrimental to individuals of low socioeconomic status, and the results also question whether justice perception reflects the true fairness of society.

## Introduction

### Social Justice Perception and Its Classification

Social justice perception refers to individuals’ evaluations of the degree of perceived social fairness; that is, individuals use “social actualities as they should be” as the criterion, and then judge fairness or lack thereof based on whether the society conforms to this criterion (Jost and Kay, 2010). Social justice perception is a subjective quality that is based on objective fairness—which is consistent with the principle of universal justice that involves equality of opportunity, fairness of rules, and equality of rights—and individuals’ perceptions of social justice are often influenced by their social perceptions (e.g., stereotypes) and values (e.g., belief in a just world; Gollwitzer and van Prooijen, 2016).

There are two approaches to studying perceived justice. The first focuses on how specific events in the environment stimulate and influence the individual’s personal experience of fairness; this is known as personal justice (Lipkus et al., 1996; Cropanzano et al., 2001). The second concerns the individual’s general justice perception, which is an overall judgment of justice that is directed toward societal entities (e.g., individuals, organizations, or society in general), and does not refer to any specific event; this is known as systemic justice (Cropanzano et al., 2001; Kay and Jost, 2003). Personal justice affects judgments of systemic justice, which indirectly affects individuals’ behaviors (Choi, 2008).

### Stereotypes of the Upper and Lower Classes: Models and Explanations

As the Internet continues to develop and with the increasing popularity of smartphones in China, certain sensitive topics involving tensions between upper- and lower-class groups have been disseminated rapidly through network interactions and have attracted public attention and discussion. However, there exists an interesting tendency whereby people differently interpret information regarding similar behaviors occurring among different groups (Cheng et al., 2012; Wei et al., 2018). For example, Chinese society is generally critical of upper-class groups, such as rich, opportunist, and corrupt officials, who many believe are callous, selfish, and lack morality. In contrast, the Chinese public is generally highly sympathetic toward lower-class groups, such as vulnerable townspeople, low-income villagers, and rural migrant workers, who many believe are warm, friendly, and kindhearted (Cheng et al., 2012; Wei et al., 2018). This polarity can be explained by the public’s stereotypes of upper- and lower-class groups, as detailed in the stereotype content model (SCM; Fiske et al., 2002). The model is based on two dimensions, namely competence and warmth, which are the two fundamental dimensions in social cognition (Abele et al., 2008; Kervyn et al., 2010; Abele and Wojciszke, 2014); in this model, high competence and high warmth are considered positive characteristics, while low competence and low warmth are considered negative characteristics. The SCM posits that the relationship between different groups within a given social structure can predict the position on each of these two dimensions of members of a given group: people of high status are considered to be highly competent, while those with low status are not; lower-class people are seen as warm, while upper-class people are seen as cold (Kervyn et al., 2010; Cheng et al., 2012; Wei et al., 2018). This theory has been tested at both explicit (Russell and Fiske, 2010) and implicit (Zhang, 2013) levels.

### Compensatory Stereotypes Affect Individuals’ Social Justice Perception

Mao Zedong, the founder of the People’s Republic of China, once uttered a widely influential sentence: “The lowly are most intelligent; the elite are most ignorant.” After analyzing Chinese left-wing films in the early 1930s, the researchers found that all the characters in the films set their characteristics and even appearance according to their social class: e.g., the rich must be portrayed with negative imagery and as bad people with obscene or ugly appearances, whereas the poor must be the positive image, the good people of beautiful or handsome appearance (Yuan, 2015). The stereotype of “the rich are bad and the poor are good” has always been deeply rooted in culture, and is more prominent on today’s Internet, television, and movies.

Therefore, such negative stereotyping of the rich and positive stereotyping of the poor in the Chinese context can promote the public’s experiences of fairness. The system justification theory (SJT) proposed by Jost et al. (2004) can explain this phenomenon. Specifically, survival in an unjust, uncontrollable, and volatile world represents an unbearable threat; thus, people tend to rationalize reality by considering the existing social division of labor as fair, legitimate, and reasonable, in order to maintain a system justification motive. One way people idealize a society is *via* compensatory stereotypes. This refers to assigning compensatory characteristics to upper- and lower-class groups, namely stereotypes that attribute upper- and lower-class groups their own unique and mutually offsetting advantages and disadvantages (Jost et al., 2004; Jost and Kay, 2005). This motivates the individual to feel that society is balanced in certain ways, and that the system is fair or at least not unbearably unfair. In literature, film, and popular culture, the poor are usually seen as happier and more honest than the rich, and reading compensatory expressions regarding the poor and the rich strengthens participants’ support of the *status quo* in society (Kay and Jost, 2003; Kervyn et al., 2010). According to SJT, compensatory stereotypes of upper- and lower-class groups (i.e., endowing the rich with negative characteristics and the poor with positive characteristics) have the psychologically significant function of maintaining justice perception. Therefore, what is noteworthy and novel is the question whether, to maintain the system justification motive, the compensatory stereotypes of upper- and lower-class groups enhance individuals’ justice perceptions. So far, there has been no relevant literature on this topic.

In addition, China is a collectivist country that is deeply influenced by Confucian culture; thus, the Chinese attach great importance not only to the warmth of individuals—expressed in characteristics such as being cooperative, friendly, coadjutant, and easygoing—but also to individual displays of competence through traits such as being modest, self-effacing, humble, and low-key. Moreover, Confucian culture emphasizes maintaining good interpersonal relationships by de-emphasizing expressions of individual abilities in order to achieve the goal of harmonious development of the society (Lim, 2009; Tong and Mitra, 2009; Luo et al., 2013). This culture tends to place more emphasis upon individual interpersonal relationships and social approval, rather than upon the role of individual competence. Therefore, a Confucian culture such as that of China pays more attention to the individuals’ characteristics of warmth rather than competence. Combining SCM with SJT enables us to deduce the following. Activating compensatory stereotypes of warmth (low-warmth rich and high-warmth poor) could enhance individuals’ perception of *systemic justice*; under the conditions of compensatory competence, the individuals’ perception of *personal justice* would be reduced because the situation of low-competence rich and high-competence poor is contrary to SCM; moreover, individuals’ *systemic justice* perception may not be affected because the Chinese culture ignores individuals’ competence. Accordingly, we propose the following hypotheses:

*Hypothesis 1*: Individuals’ perception of social justice (personal justice and systemic justice) in a compensatory warmth condition is higher than that in a non-compensatory warmth condition.

*Hypothesis 2*: Individuals’ personal justice perception in a compensatory competence condition is lower than that in a non-compensatory competence condition; moreover, the systemic justice perception is unchanged.

Hence, this study recruited college students, who were presented with vignettes to explore the impact on their justice perception of assigning positive characteristics to the poor and negative characteristics to the rich.

## Materials and Methods

### Participants

A total of 339 undergraduate or graduate students were collectively tested in classrooms. Of the participants, 159 were male (46.9%) and 180 were female (53.1%). Ages ranged from 17 to 30 years, with an average of 22.37 years (SD = 1.98). The participants were randomly assigned to different experimental treatments, and the number of participants in each group was kept as consistent as possible.

The socioeconomic status (SES) of the participants was assessed using the MacArthur Scale of Subjective SES (Adler et al., 2000). The participants were given a drawing of a ladder with 10 rungs and asked to imagine that the ladder represented individuals’ social class status. The higher the rung, the higher the SES. They were then asked to place an *X* on the rung that best represented where they believed they stood on the ladder. There is no outlier among the results, and the mean value was moderately anchored (*M* = 5.39, SD = 0.97).

### Materials

Based on the SCM, this study used vignettes to manipulate the positive and negative aspects of two fundamental dimensions: competence and warmth. The SCM itself can be considered a compensatory model, and the relationship between the two dimensions of competence and warmth is often mixed; that is, it is often observed that a high evaluation on one dimension is paired with a low evaluation on the other dimension. This compensatory relationship between the two dimensions of competence and warmth has been validated in studies of both virtual and actual groups (Cuddy et al., 2007; Cheng et al., 2012). To prevent mutual interference between the two dimensions, competence and warmth were controlled by being rated separately.

A 2 × 2 between-subjects design manipulated the participants’ perceptions toward the target characters on two dimensions: a characteristic dimension (competence–warmth) and an activation condition (compensatory–non-compensatory). The dependent variables were personal justice perception and systemic justice perception. The participants were randomly assigned to one of four conditions, in which they were presented with different descriptions regarding the character in the vignettes: high-competence rich and low-competence poor (non-compensatory competence); low-competence rich and high-competence poor (compensatory competence); high-warmth rich and low-warmth poor (non-compensatory warmth); low-warmth rich and high-warmth poor (compensatory warmth).

#### Materials for Activating Compensatory Stereotypes

The participants were asked to read vignettes about two target characters named Li Jun and Wang Tao; each vignette reflected a certain characteristic dimension. There were two scenarios: one in which the rich individual’s behavior was positive and the poor individual’s behavior was negative, depicting non-compensatory stereotypes, and the other where the rich individual’s behavior was negative and the poor individual’s behavior was positive, illustrating compensatory stereotypes. Li Jun was described as of high SES and Wang Tao as of low SES. Each was described in a given vignette as high-competence, low-competence, high-warmth, or low-warmth. For example, the vignettes expressing compensatory competence (low-competence rich, high-competence poor) were as follows:

Li Jun, a 40-year-old senior manager of an investment bank, lives in a luxury villa in the center of an international metropolis. He owns several properties, and his daughter is studying in the best schools abroad. He and his wife often travel around the world on private planes for holidays. However, he has been making constant mistakes at work recently, and many parts of business have done very badly, so that his supervisors and colleagues are beginning to think that his abilities are not up to the current job.

Wang Tao is a 40-year-old cleaner in a bus company. There are three people in his family, who live in an old house 10 square meters in size. He works very hard, but his life is very difficult, and his meager salary merely keeps his family from going hungry. He is always worrying about paying for his children’s tuition when the new semester begins. However, he has done well in his work, even when faced with many difficult tasks, so that his supervisors and colleagues have praised him for his abilities.

In the non-compensatory competence vignette, Wang Tao had high-competence while Li Jun had low-competence. As such, the characterizations of the two characters were reversed across vignettes; the wording was slightly modified to ensure fluency in each case. Similarly, the wording of the compensatory/non-compensatory warmth vignette changed the characterization from competence to warmth.

#### Scales for Measuring Justice

The System Justification Scale compiled by Kay and Jost (2003) and the Just World Scale for Self compiled by Lipkus et al. (1996) were modified and used in this study. Each scale consists of eight items, with responses collected using a 7-point Likert-type scale ranging from 1 (*greatly disagree* or *totally inapplicable*) to 7 (*greatly agree* or *totally applicable*).

A pilot study was conducted before the scale was implemented in the formal experiment. An exploratory factor analysis with a sample size of 274 was used to reduce the items down to a factorial structure. The method of principal axes with oblique rotation was used for factor extraction, as illustrated in Table 1. Factors with an eigenvalue greater than 1 were extracted, while items with a factor loading greater than 0.3 were retained. A total of 13 items that encompassed two factors were extracted, which accounted for 55.38% of the variance. Referring to the original theoretical constructs and analyzing the theoretical significance of the items, factor 1 (seven items in total) was named personal justice perception, that is, the individual’s personal experience of fairness. Example items are “I feel that I earn the rewards and punishments I get” and “I feel that when I meet with misfortune, I have brought it upon myself.” Factor 2 (six items in total) was named *systemic justice perception*, as it referred to perceptions of the fairness, legitimacy, and justifiability of the prevailing social system. Sample items included sentiments such as “Everyone has a fair shot at wealth and happiness” and “Society is set up so that people usually get what they deserve.” The coefficients of internal consistency of the two dimensions were 0.89 and 0.83, respectively.

**Table 1:** Exploratory factor analysis of the scales for measuring justice perception (*N* = 274).

Item	Factor 1: personal justice perception	Factor 2: systemic justice perception	Communality
I feel that I get what I deserve	0.78		0.71
I feel that the world treats me fairly	0.76		0.77
I feel that my efforts are noticed and rewarded	0.69		0.72
I feel that people treat me fairly in life	0.65		0.73
I feel that people treat me with the respect I deserve	0.57		0.68
I feel that I earn the rewards and punishments I get	0.55		0.65
I feel that when I meet with misfortune, I have brought it upon myself	0.48		0.59
In general, you find society to be fair		0.74	0.74
Most policies serve the greater good		0.68	0.58
Our society needs to be radically restructured (reverse-scored)		0.65	0.72
Everyone has a fair shot at wealth and happiness		0.62	0.69
Our society is getting worse every year (reverse-scored)		0.54	0.61
Society is set up so that people usually get what they deserve		0.38	0.69
Cronbach’s alpha	0.89	0.83	
Eigenvalue	9.27	1.65	
Variance explained, %	39.25	16.12	
Cumulative variance	39.25	55.38	

KMO = 0.87; Bartlett test of sphericity = 2312.78, p < 0.001.

### Procedure

The participants were informed that they were to participate in two experiments. First, compensatory or non-compensatory stereotypes were activated. The participants were informed that the purpose of the first experiment was to study the mechanism of impression formation of strangers. The participants read the passages about the target characters. The validity of the experimental manipulation (activation of the stereotypes) was then checked using a single item. To assess the participants’ perception of the group to which each of Li Jun and Wang Tao belonged, they were asked “In your opinion, what is the socioeconomic status of Li Jun/Wang Tao in the society?”; responses were collected using a 7-point scale that ranged from 1 (*poor*) to 7 (*rich*). The participants were then asked to assess the personality characteristics of Li Jun and Wang Tao in terms of four adjectives that described the dimensions of competence (i.e., competent, capable) and warmth (i.e., warm, friendly) of the SCM (Cuddy et al., 2007), using a scale from 1 (*totally inapplicable*) to 7 (*totally applicable*).

Second, justice perception was measured. The participants were informed that the purpose of the second experiment was to assess the individual’s views and attitudes toward social issues. They completed a package of questionnaires, including the Personal Justice Perception Questionnaire and the System Justice Perception Questionnaire. After finishing the experiment, the participants were given a small reward and asked if they had any questions about the purpose of the study.

## Results

### Manipulation Check

#### Perception of Target Character’s Socioeconomic Status

Fourteen participants (11 male and 3 female) wrongly perceived the SES of the target character, by assigning a rating of less than 5 points to Li Jun or higher than 3 points to Wang Tao. After eliminating these participants, 325 remained. The Kendall’s coefficient of concordance of inter-rater reliability was 0.92, *χ*^2^(324) = 379.79, *p* < 0.01. The mean rating of the SES of Li Jun was *M* = 5.91 (SD = 0.54), while that for Wang Tao was 1.72 (SD = 0.60); the difference between ratings was significant, *t*(323) = 73.66, *p* < 0.001, *d* = 8.34. Thus, the SES of the target characters was successfully manipulated.

#### Manipulation Check of the Activation Condition

We ran a two-way mixed ANOVA to test the validity of stereotype activation for the competence and warmth ratings. The between-subject variable was the activation condition (compensatory vs. non-compensatory stereotype), and the within-subject variable was the group to which the target character (Li Jun or Wang Tao) in the vignette belonged (the rich vs. the poor). For the competence ratings, the competence score was the dependent variable. Results showed a significant interaction between group and the activation condition, *F*(1, 162) = 213.14, *p* < 0.001, *η*^2^ = 0.42. The main effect of group was also significant: the evaluation of the competence of the rich character (*M* = 5.46, SD = 1.07) was significantly higher than that of the poor character (*M* = 3.34, SD = 1.20), *F*(1, 162) = 336.52, *p* < 0.001, *η*^2^ = 0.39. A further simple-effects analysis found that in the non-compensatory condition, the participants’ competence evaluation of the rich character (*M* = 6.17, SD = 0.53) was significantly higher than that of the poor character (*M* = 2.42, SD = 0.76), *F*(1, 82) = 472.69, *p* < 0.001, *η*^2^ = 0.54, while in the compensatory condition, there was no significant difference between the participants’ competence evaluation of the rich character (*M* = 4.47, SD = 0.82) and that of the poor character (*M* = 4.36, SD = 0.78), *F*(1, 79) = 0.82, *p* = 0.382, *η*^2^ = 0.02. This confirms that compensatory/non-compensatory stereotypes were successfully activated for the competence ratings.

For the warmth ratings, the warmth score was analyzed as the dependent variable. Similarly, the interaction between group and the activation condition was significant, *F*(1, 161) = 92.12, *p* < 0.001, *η*^2^ = 0.16. The main effect of group was not significant; there was no significant difference between the evaluation of the warmth of the rich character (*M* = 3.91, SD = 0.95) and that of the poor character (*M* = 4.03, SD = 1.26), *F*(1, 161) = 0.98, *p* = 0.332, *η*^2^ = 0.03. A further simple-effects analysis revealed that in the compensatory condition, the participants’ warmth evaluation of the rich character (*M* = 3.39, SD = 0.81) was significantly lower than that of the poor character (*M* = 4.95, SD = 1.03), *F*(1, 80) = 58.46, *p* < 0.001, *η*^2^ = 0.13, while in the non-compensatory condition, the warmth evaluation of the rich character (*M* = 4.29, SD = 0.90) was significantly higher than that of the poor character (*M* = 3.09, SD = 0.69), *F*(1, 80) = 58.46, *p* < 0.001, *η*^2^ = 0.11. Hence, the manipulation of compensatory/non-compensatory stereotypes was successful in terms of warmth.

### Effect of Activating Compensatory Stereotypes on Personal Justice Perception and Systemic Justice Perception

**Figure 1:**
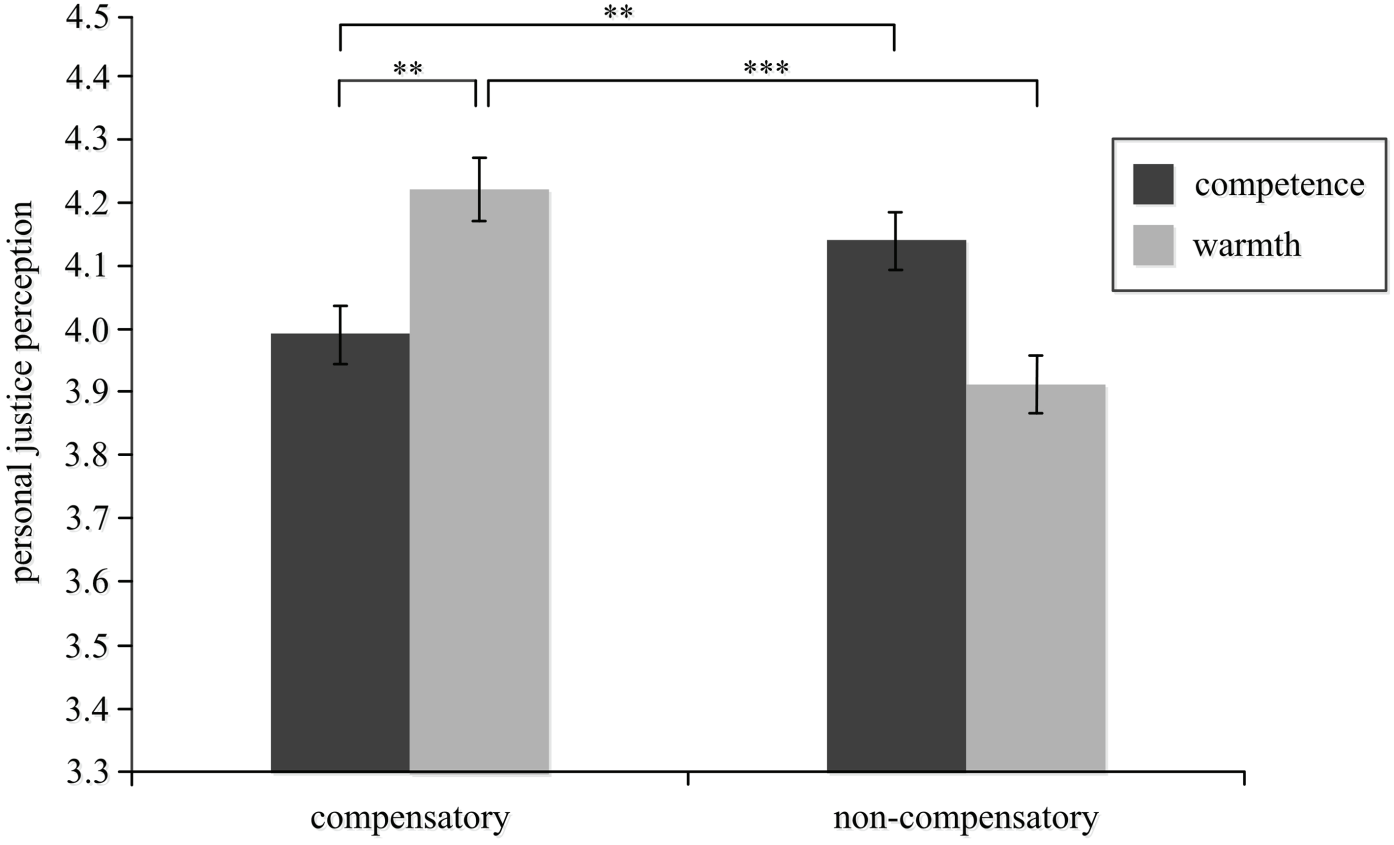
Effects of compensatory stereotypes on different dimensions on personal justice perception. **p < 0.01, ***p < 0.001.

Analysis of variance was performed using the characteristic dimension (i.e., competence and warmth) and the activation condition as independent variables and personal justice perception ratings as the dependent variable. The main effect of the activation condition was not significant, *F*(1, 323) = 4.93, *p* = 0.077, *η*^2^ = 0.06, neither was the main effect of the characteristic dimension, *F*(1, 323) = 2.93, *p* = 0.113, *η*^2^ = 0.04. However, the interaction between the activation condition and characteristic dimension was significant, *F*(1, 323) = 5.79, *p* < 0.05, *η*^2^ = 0.21, as illustrated in Table 2 and Figure 1.

**Table 2:** Social justice perception scores after activating compensatory stereotypes for different dimensions.

Dimension	Compensatory or non-compensatory	*N*	Personal justice perception		Systemic justice perception	
			*M*	SD	*M*	SD
Competence	Compensatory	80	3.99	0.752	3.90	0.879
	Non-compensatory	83	4.14	0.832	3.97	0.881
	Total	163	4.08	0.818	3.94	8.876
Warmth	Compensatory	81	4.22	0.877	3.99	0.831
	Non-compensatory	81	3.91	0.727	3.38	0.887
	Total	162	4.13	0.860	3.71	0.912

A further simple-effects analysis revealed that in the non-compensatory stereotype condition, there was no significant difference between the two levels of the characteristic dimension, *F*(1, 163) = 0.51, *p* = 0.428, *η*^2^ = 0.05. In other words, when the positive characteristics of the rich and the negative characteristics of the poor were activated, there was no effect on personal justice perception among the participants. In the compensatory stereotype condition, the difference between the two levels of the characteristic dimension was significant, *F*(1, 160) = 7.38, *p* < 0.01, *η*^2^ = 0.18. The participants who had activated the “low-warmth rich” and “high-warmth poor” stereotypes had higher personal justice perception than those who had activated the “low-competence rich” and “high-competence poor” stereotypes. Regarding the characteristic dimension of competence, there was a significant difference between compensation level and non-compensation level, *F*(1, 162) = 11.08, *p* < 0.01, *η*^2^ = 0.21. The participants who had activated the “low-competence rich” and “high-competence poor” stereotypes had lower perceived personal justice than those who had activated the non-compensatory stereotypes. Regarding the characteristic dimension of warmth, there was also a significant difference between compensation level and non-compensation level, *F*(1, 161) = 13.27, *p* < 0.001, *η*^2^ = 0.29. The participants who demonstrated the “low-warmth rich” and “high-warmth poor” stereotypes had higher personal justice perception than those who demonstrated the “high-warmth rich” and “low-warmth poor” stereotypes.

**Figure 2:**
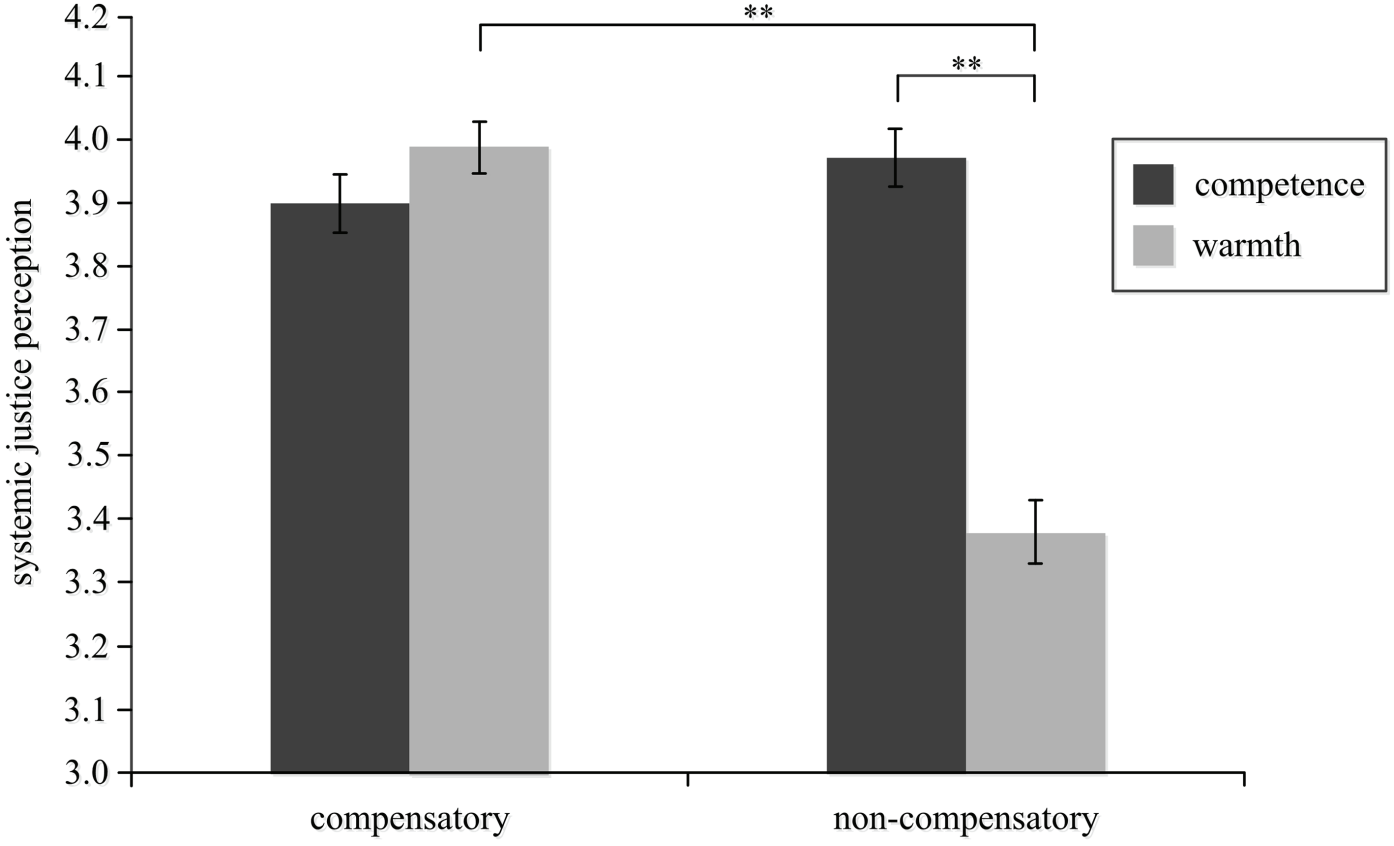
Effects of compensatory stereotypes on different dimensions on systemic justice perception. **p < 0.01.

Analysis of variance was performed using the characteristic dimension and activation condition as independent variables, and systemic justice perception as the dependent variable. The main effect of the activation condition was not significant, *F*(1, 323) = 3.37, *p* = 0.081, *η*^2^ = 0.02, neither was the main effect of the characteristic dimension, *F*(1, 323) = 2.78, *p* = 0.098, *η*^2^ = 0.03. However, the interaction between the activation condition and characteristic dimension was significant, *F*(1, 323) = 5.83, *p* < 0.05, *η*^2^ = 0.14, as illustrated in Table 2 and Figure 2.

A further simple-effects analysis revealed that for the compensatory stereotype condition, there was no significant difference between the two levels of the characteristic dimension, *F*(1, 160) = 0.44, *p* = 0.519, *η*^2^ = 0.05. When the negative characteristics of the rich and the positive characteristics of the poor were activated, there was no effect on systemic justice perception among the participants. In the non-compensatory stereotype condition, the difference between the two levels of the characteristic dimension was significant, *F*(1, 163) = 6.93, *p* < 0.01, *η*^2^ = 0.37. The participants who had activated the “high-warmth rich” and “low-warmth poor” stereotypes had lower systemic justice perception than those who had activated the “high-competence rich” and “low-competence poor” stereotypes. Regarding the characteristic dimension of warmth, there was a significant difference between compensation level and non-compensation level, *F*(1, 161) = 10.11, *p* < 0.01, *η*^2^ = 0.45. The participants who had activated the “high-warmth rich” and “low-warmth poor” stereotypes had lower perceived systemic justice than those who had activated the non-compensatory stereotypes.

## Discussion

The findings verified the hypothesis we proposed at the beginning of the study. That is, activating compensatory stereotypes of warmth (i.e., the rich exhibit low warmth and the poor exhibit high warmth) could enhance individual perceptions of personal justice and systemic justice, while activating compensatory stereotypes of competence (i.e., the rich exhibit low competence and the poor exhibit high competence) could weaken individual perceptions of personal justice, but not change their perceptions of systemic justice. Hence, we can affirm the title of our paper that perceiving the poor as warm and the rich as cold enhances perceived social justice.

Under the characteristic dimension of warmth, the participants who demonstrated compensatory stereotypes of warmth (i.e., the rich with low warmth and the poor with high warmth) reported higher perceived social justice (personal justice and systemic justice) than those who demonstrated non-compensatory stereotypes of warmth. Therefore, *Hypothesis 1* is verified. More specifically, demonstrating the stereotypes that the rich are warm and the poor are cold reduced systemic justice perception among the participants. Fiske et al. (2007) found that collectivistic cultures emphasize warmth but individualistic cultures emphasize competence. In a collectivistic culture such as China, the poor have limited survival resources and must gain resources and overcome difficulties through receiving interpersonal mutual assistance (warm orientation). In contrast, the rich have abundant resources, pay more attention to competence, and expend less effort on unnecessary interpersonal communications (competent orientation), consistent with the conclusion of Wei et al. (2018). Therefore, activated compensatory stereotypes of warmth could enhance individuals’ perception of personal justice and systemic justice. These observations are inconsistent with the theory that coldness in the rich will lead to low justice perception, and thus, reduce systemic justice perception (Henry and Saul, 2006); rather, they support SJT (Jost et al., 2004).

Under the characteristic dimension of competence, the participants who demonstrated compensatory stereotypes of competence reported lower perceived personal justice than those who demonstrated non-compensatory stereotypes of competence; moreover, such perception did not affect the systemic justice perception of the participants. Thus, *Hypothesis 2* is also validated. In addition, the participants who had activated compensatory stereotypes of warmth had higher personal justice perception than those who had activated compensatory stereotypes of competence. In the competence compensation conditions, the rich individuals were described as having more resources and higher social status despite their low competence, while the poor individuals had higher competence, but fewer resources and lower social status; this led the participants to generate lower ratings of personal justice perception. This extends Kay and Jost’s (2003) research findings that people judge social justice based on their own experiences (Zhou and Guo, 2013): when the activation of a compensatory stereotype is inconsistent with their own experience (e.g., the poor are of low competence and the rich are of high competence), people’s perceptions of personal justice reduce.

Meanwhile, this phenomenon is deeply influenced by the *guanxi* orientation in Chinese Confucian culture—that is, Chinese people attach importance to establishing harmonious relationships between people from an early age rather than displaying individual abilities. Within this worldview, modesty is regarded as a virtue in interpersonal communication (Tong and Mitra, 2009; Luo et al., 2013). This culture tends to place more emphasis on individual interpersonal relationships and social approval rather than upon individual competence. Hence, activating the non-compensatory stereotypes of competence did not affect the systemic justice perception of the individuals; meanwhile, the individuals who had activated compensatory stereotypes of warmth had higher personal justice perception than those who had activated compensatory stereotypes of competence.

There is also the fact that participants who had activated non-compensatory stereotypes of warmth displayed lower systemic justice perception than those who had activated non-compensatory stereotypes of competence. The rich, as a group with higher social status, are inherently stereotyped as having strong competence (Wei et al., 2018), and thus, manipulating this dimension has little effect on subsequent systemic justice perception. However, if the rich possess both wealth and good interpersonal relationships, then individuals could perceive that the whole culture or the whole world is unreasonable. Hence, the participants perceived systemic justice as poorer in the non-compensatory condition, regarding the warmth dimension (but not the competence dimension).

Thus, in a culture that emphasizes that collective relationships are more important than personal abilities, the public endowed different groups with compensatory stereotypes in order to maintain overall balance, such as “the rich are cold” and “the poor are warm.” This supports the idea that the system justification motive can serve as a personal resource and reduce the perceived negative effects of injustice, thus preserving mental health (Furnham, 2003). Although previous studies by Chinese researchers did not directly confirm this particular cause and effect relationship, related studies can be used to support this proposition. For example, Weng (2010) showed that people with perceptions of injustice tend to have a social attitude of “sympathizing with the poor and despising the rich.” As the degree of injustice increases, hostility toward the rich is more easily triggered. Based on previous studies using data from the Chinese General Social Survey, Li et al. (2012) confirmed that there are close relationships among perceived justice regarding income, the distribution of life opportunities, and social conflict awareness: the greater the perceived injustice, the greater the awareness of social conflict. Moreover, the behaviors from the intergroup affect and stereotypes map (BIAS Map; Cuddy et al., 2007), which is a modified version of the SCM, suggest that different warmth and competence combinations in social interactions correspond to different emotional and behavioral tendencies. Rich groups are perceived as high-competence and low-warmth, which triggers individuals’ envy and elicits passive facilitation but active harm. The perception of poor groups, meanwhile, as having low competence and high warmth, triggers individuals’ pity and elicits active facilitation but passive harm. Envy and pity often arise from social comparative situations and are closely related to the acquisition, distribution, and prioritization of resources (Lu et al., 2013; Zhang et al., 2017). From the perspective of psychological processes and motivation, this signifies that a greater perception of injustice endows the rich with a more negative evaluation or expands the differences between the rich and poor, thereby producing a buffering effect that reduces emotional stress associated with social injustice.

The results of this study illustrate that the perception of justice is impacted by compensatory stereotypes triggered by the system justification motive. Additionally, in a related point, numerous compensatory stereotypes are presented in Chinese films and television programs (e.g., that the poor are warm and helpful and the rich are indifferent and selfish; Yuan, 2015). These films and television programs not only enhance the perception of social justice of the audience, but also shape their knowledge and experience. Therefore, compensatory stereotypes activated by such films and television shows may be both beneficial and detrimental to low-class groups. From one perspective, the perceived social justice engendered by compensatory stereotypes can act as a psychological resource that helps individuals of low SES to cope with threats, obtain positive psychological states (Correia et al., 2009; Xie et al., 2011), and promote the attainment of long-term goals (Hu et al., 2016). In contrast, the perceived social justice engendered by this compensatory stereotype encourages individuals of low SES to defend the injustice of the social system (Henry and Saul, 2006; Beierlein et al., 2011). This is neither conducive to the change of status and upward mobility of individuals with low SES (Zhou and Guo, 2013), nor to the promotion of social justice governance (Gaucher et al., 2010). In other words, on the one hand, compensatory stereotypes make lower-class groups embrace the status quo and refuse to change; on the other hand, various objective injustices make upward mobility impossible for them, regardless of how hard they work. Once certain sensitive topics involving tensions between upper- and lower-class groups are disseminated, lower-class groups can only maintain the perceived social justice by endowing higher-class groups with more negative characteristics, and thus, hostility toward the rich emerges as time passes. Of course, it is not conducive to social stability and progress. In addition, this study also questions the view that social justice perception can largely reflect the true degree of social justice (Jost and Kay, 2010; Hu et al., 2016).

Although the current study revealed interesting findings, it also has the following limitations. First, the experimental paradigm used in this study measured justice perception explicitly, but explicit justice perception may be affected by factors such as social approval. Implicit measurements should be adopted in the future. Second, competence and warmth are different in terms of social desirability, which should be considered in future research.

## Conclusion

Compensatory stereotypes triggered by system justification motives can affect the social justice perceptions of individuals to a certain extent. That is, perceiving the poor as warm and the rich as cold enhances perceived social justice, whereas perceiving the poor as competent and the rich as incompetent reduces perceived personal justice, but does not affect perceived systemic justice. Especially in the context of the Chinese Confucian culture, which emphasizes warmth but ignores competence, the effect of compensatory stereotypes on perceptions of social justice underscores a cultural difference with the West that warmth is superior to competence. In addition, compensatory stereotypes may be either beneficial for or detrimental to individuals of low socioeconomic status.

## Data Availability

The datasets for this manuscript are not publicly available because the raw data supporting the conclusions of this manuscript will be made available by the authors, without undue reservation, to any qualified researcher. Requests to access the datasets should be directed to island4u@foxmail.com.

## Ethics Statement

This study was carried out in accordance with the recommendations of the Tianjin University Research Ethics Committee. The protocol was approved by the Tianjin University Institutional Review Board. Participants gave written informed consent in accordance with the Declaration of Helsinki.

## Author Contributions

AF designed the experiments, analyzed the data, and wrote the manuscript. ZZ designed the experiments and worked on the final version of the manuscript. WH and ZL contributed to the analysis and interpretation of the data. NW, GH and TY collected the data and contributed with additional writing.

### Conflict of Interest Statement

The authors declare that the research was conducted in the absence of any commercial or financial relationships that could be construed as a potential conflict of interest.
